# Urban Recreation Areas as Foci of Tick Hazard: Multi-Year Seasonal Patterns of *Ixodes ricinus* and *Dermacentor reticulatus* Activity and Host Spectrum of Their Juvenile Stages in Eastern Poland

**DOI:** 10.3390/biology15030252

**Published:** 2026-01-29

**Authors:** Zbigniew Zając, Aneta Woźniak, Joanna Kulisz

**Affiliations:** Department of Biology and Parasitology, Medical University of Lublin, Radziwiłłowska 11, 20-080 Lublin, Polandaneta.wozniak@umlub.edu.pl (A.W.)

**Keywords:** *Ixodes ricinus*, *Dermacentor reticulatus*, juvenile ticks, *Apodemus agrarius*, rodents, ticks in urban areas, tick-host interactions

## Abstract

Urban green spaces increasingly serve as sites of human–tick contact, yet long-term data on tick activity and host associations in urban recreational areas remain limited. This study investigated the seasonal activity patterns of *Ixodes ricinus* and *Dermacentor reticulatus* and the host spectrum of juvenile tick stages in an urban park in eastern Poland over a five-year period (2015–2019). Questing ticks were collected from vegetation using the flagging method, while small mammals were live-trapped to assess tick infestation of juvenile stages. The effects of air temperature, relative humidity, and seasonality on tick activity were analysed using generalized additive models (GAMs). *D. reticulatus* was the dominant tick species throughout the study, exhibiting pronounced autumn activity peaks, whereas *I. ricinus* occurred at lower densities with peak activity in late spring and early summer. GAM analyses revealed that apparent temperature effects observed in uncorrected models disappeared after accounting for seasonality, while seasonal timing remained a strong and consistent predictor of tick activity across species, developmental stages, and sexes. Juvenile ticks of both species were most frequently associated with *Apodemus agrarius*, indicating that urban-adapted rodent hosts play a key role in sustaining tick life cycles in simplified urban ecosystems. These findings demonstrate that urban recreational areas can function as persistent foci of tick hazard, with tick activity driven primarily by intrinsic seasonal dynamics rather than short-term weather variation.

## Simple Summary

Urban parks and green spaces are widely used for recreation but may also increase human exposure to ticks. In this study, tick activity was monitored over five years in urban park in eastern Poland. Two common European species, *Dermacentor reticulatus* and *Ixodes ricinus*, were examined to describe their seasonal activity and host associations. *D. reticulatus* was more abundant and showed pronounced autumn activity, whereas *I. ricinus* was most active in late spring and early summer. Tick activity was primarily related to seasonal timing rather than short-term variation in temperature or humidity. Juvenile stages of both species were most frequently found on striped field mice (*Apodemus agrarius*), highlighting the role of urban-adapted rodents in maintaining tick populations. These results indicate that urban recreational areas can act as persistent sources of tick exposure with predictable seasonal risk.

## 1. Introduction

Tick-borne diseases (TBDs) represent an emerging public health challenge in urban green spaces, where human exposure to ticks has increased as a result of growing recreational use [1]. Urban parks, forests, and other components of green infrastructure provide suitable habitats for both wildlife and ticks, facilitating the introduction and maintenance of tick-borne pathogens (TBPs) within urban environments [2,3]. Consequently, viable tick populations are now established in many urban green areas, often exhibiting infection rates (e.g., *Borrelia* spp.) comparable to those recorded in adjacent rural habitats, which may offset the public health benefits of urban nature by increasing residents’ exposure to TBDs [2,4,5]. In this context, ixodid ticks of the genera *Ixodes* and *Dermacentor* are increasingly reported in cities; *Ixodes ricinus* acts as the principal European vector of *Borrelia* spp. causing Lyme borreliosis (LB) and tick-borne encephalitis virus (TBEV), while *Dermacentor reticulatus* transmits pathogens such as *Babesia canis*, *Rickettsia* spp. [6,7,8]. Both species predominate in Poland and have expanded their distribution ranges over recent decades, including progressive encroachment into suburban and urban habitats [9,10,11,12,13,14,15,16,17].

Poland’s Lublin region offers a pertinent case study of urban tick ecology. Eastern Poland harbours one of the highest tick densities nationwide, and both *I. ricinus* and *D. reticulatus* are abundant in the region’s forests and meadows. *I. ricinus* typically thrives in moist, shaded woodlands, whereas *D. reticulatus* (the “meadow tick”) favours open habitats like grasslands and riparian meadows [13,14,15,16]. Nonetheless, these species often co-occur in ecotones and peri-urban areas, even parasitizing the same hosts [18].

Climate warming and land-use changes have facilitated the expansion and increased activity of ticks in urban and peri-urban environments, where milder winters have extended the seasonal window of questing activity [19,20]. Although multi-year observations reveal pronounced interannual variability and bimodal activity patterns in *D. reticulatus*, and a late spring–early summer activity peak in *I. ricinus*, the extent to which short-term weather conditions drive tick activity independently of intrinsic seasonal regulation remains poorly resolved [13,21,22]. Moreover, despite the widespread presence of simplified rodent communities in urban green spaces [3,23,24], there is a marked lack of integrated studies linking tick seasonal dynamics with the identity and relative importance of urban-adapted rodent hosts sustaining juvenile tick stages and TBPs.

Therefore, the aim of this study was to quantify the seasonal activity patterns of *I. ricinus* and *D. reticulatus* in an urban recreational area based on multi-year field data, and to disentangle the relative contributions of short-term weather conditions and intrinsic seasonal regulation to tick questing activity. In parallel, we sought to identify the host spectrum of juvenile tick stages in this urban environment, with particular emphasis on the role of dominant urban-adapted rodent species in sustaining local tick populations. We hypothesized that seasonal timing exerts a stronger influence on the questing activity of *I. ricinus* and *D. reticulatus* than short-term variation in temperature and relative humidity, that seasonal activity patterns differ between species, developmental stages, and sexes, and that juvenile stages of both tick species are predominantly associated with a limited number of urban-adapted rodent hosts, reflecting a simplified host–tick system in urban environments.

## 2. Materials and Methods

### 2.1. Study Area

Field studies were conducted within the city of Lublin (eastern Poland), in its northern part, in an area that formerly served as a military training ground and has subsequently undergone a process of ecological restoration. At present, the site functions as an urban park and is widely used for recreational purposes by local residents.

**Figure 1:**
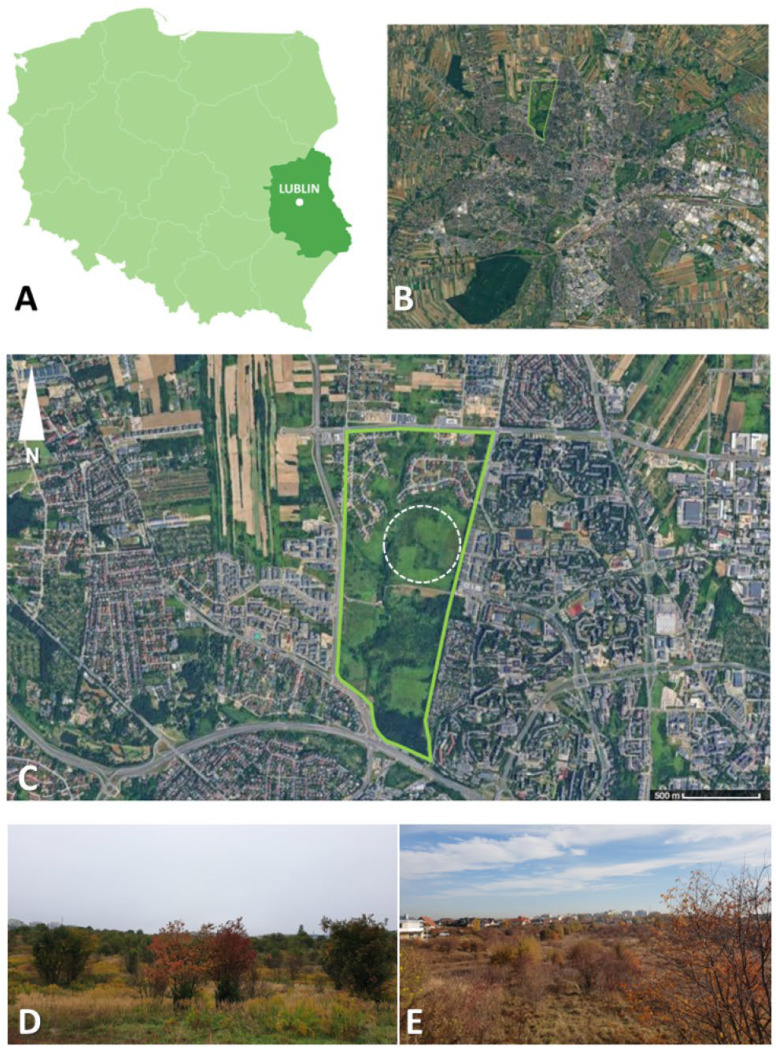
Study area. Panel (**A**) shows the location of the city of Lublin within Poland and the Lublin Voivodeship. Panels (**B**,**C**) depict the study area, outlined by a white dashed line, located in an enclave of urban greenery within the city and highlighted in bright green. Panels (**D**,**E**) illustrate the ecosystem of the study area, characterized by herbaceous plants, grasses, shrubs, and small isolated trees, with surrounding urban architecture visible in the background. The figure was prepared using the datawrapper.de platform and based on data from Google Maps. Photos in panels (**D**,**E**) were taken by Z. Zając.

The study area covers a relatively large surface and constitutes a distinct ecological enclave surrounded by dense urban development (Figure 1). The total area of this enclave is approximately 100 ha; within its central part, a designated sampling plot was established, where ticks were systematically collected for 30 min during each sampling event. The vegetation is predominantly meadow-type, with an ongoing process of ecological succession manifested by the patchy occurrence of shrubs and scrub vegetation. In recent years, extensive parts of the area have become dominated by the invasive plant species Canadian goldenrod (*Solidago canadensis*).

The region is characterized by a temperate continental climate, with distinct seasonal variation. Mean annual air temperature is approximately 10 °C, with cold winters and warm summers. Precipitation is moderate and unevenly distributed throughout the year, with the highest rainfall occurring during late spring and summer. Snow cover and sub-zero temperatures typically occur during winter months, which limits tick activity during this period [21,25].

### 2.2. Tick Collection

Ticks were collected from vegetation using the flagging method, which involved sweeping the vegetation with a white flannel cloth of approximately 1 m^2^ surface area. After traversing a transect of approximately 10 m, the cloth was inverted and all attached ticks were carefully removed and transferred into plastic containers.

Field surveys were conducted over five consecutive years (2015–2019). During each time, tick collection was performed for a standardized duration of 30 min. Sampling events were planned, whenever possible, at regular time intervals (on average every 3 weeks) and carried out only under favourable weather conditions, excluding days with precipitation, strong wind, snow cover, or frost.

In addition, ambient weather conditions were recorded by measuring air temperature and relative humidity at approximately 10 cm above ground level using.

Following field collection, ticks were transported to the laboratory, where they were identified to species level and developmental stage using standard morphological identification keys [26].

### 2.3. Tick-Host Spectrum

Rodents were captured in 2018, from late spring to late autumn. The trapping period was selected to minimize stress to animals confined in traps, particularly stress associated with low ambient temperatures, which could negatively affect animal welfare.

Rodent trapping was conducted using Smart VACO Pro (VACO, Domasław, Poland) live traps. During each trapping session, 50 traps were deployed in the field and placed at intervals of approximately 1 m. Traps were baited with a mixture of sunflower seeds and cereal grains. Additionally, to account for the potential incidental capture of carnivorous or omnivorous species, small portions of minced pork meat were also placed in the traps.

Traps were inspected regularly, and captured rodents were carefully removed to minimize handling time and stress. Each individual was examined immediately in the field. The entire body surface of captured rodents was thoroughly inspected, with particular attention paid to typical tick attachment sites, including the head, ears, neck, axillary regions, and groin.

All ticks collected from rodents were carefully removed using fine forceps and transferred into Eppendorf-like tubes (Googlab, Rokocin, Poland) containing 70% ethanol. In the laboratory, ticks were identified to species level and developmental stage using standard morphological identification keys [26].

All trapping and handling procedures were carried out in accordance with applicable animal welfare regulations. The study protocol was reviewed and approved by the Local Ethical Committee for Animal Experiments at the University of Life Sciences in Lublin (approval no. 72/2018).

### 2.4. Statistical Analysis of Environmental Effects on Tick Activity

Tick seasonal activity was analysed using generalized additive models (GAMs) fitted with a negative binomial error distribution to account for overdispersion in count data. Analyses were performed separately for each species and developmental group, including adult females and males of *I. ricinus* and *D. reticulatus*, as well as nymphs of *I. ricinus*. Total tick counts were not included in the analyses because they represent an aggregate measure combining individuals from different developmental stages and sexes, each of which may respond differently to environmental drivers.

Two complementary GAM frameworks were applied to evaluate the effects of environmental variables on tick activity. In the first framework, the effects of the air temperature and relative air humidity were assessed without explicitly modelling seasonal variation, where *year* was included as a categorical covariate to control for interannual variability in sampling effort and background population dynamics. In the second framework, seasonality in tick activity was explicitly incorporated to disentangle the independent effects of temperature and humidity from seasonal dynamics. Seasonal variation was modelled as a smooth function of the day of year (DOY).

The statistical significance of individual predictors was assessed using likelihood ratio tests (LRTs) based on differences in model deviance (ΔDeviance). For each predictor, the full GAM was compared with a reduced GAM in which the focal term was removed while all remaining terms were retained. Temperature and humidity effects were tested using single-degree-of-freedom comparisons, whereas the seasonal smooth term was evaluated using a multi-degree-of-freedom comparison corresponding to the effective degrees of freedom of the spline. Statistical significance was inferred at *p* < 0.05.

All GAMs were fitted independently for each species–stage–sex group to avoid pseudo-replication and to allow for group-specific responses to environmental conditions. Model estimates are reported as partial effect sizes (β) on the log scale, and graphical outputs present estimated effects together with LRT-derived *p*-values. All statistical analyses were conducted in the R software (version 4.0 for Windows; R Foundation for Statistical Computing, Vienna, Austria) using the *mgcv* package.

### 2.5. Statistical Analyses of Rodent-Associated Tick Infestation

Differences in infestation prevalence among rodent species were tested using chi-square tests of independence, separately for *I. ricinus* and *D. reticulatus*, with larval and nymphal stages pooled. Sex-specific differences in tick infestation prevalence in *A. agrarius* were tested using Fisher’s exact test, with larval and nymphal stages pooled across tick species.

## 3. Results

### 3.1. Seasonal Activity of Questing Ticks

**Figure 2:**
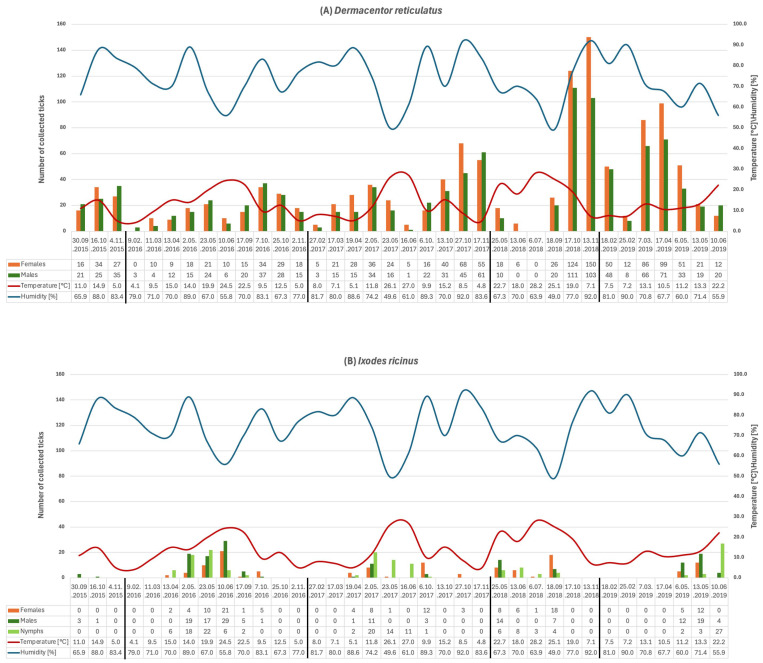
Seasonal activity of *Dermacentor reticulatus* (panel **A**) and *Ixodes ricinus* ticks (panel **B**) in relation to temperature and relative humidity.

Across the 2015–2019 sampling period, *D. reticulatus* ticks were dominant in vegetation collections compared with *I. ricinus* (2191 to 422 collected specimens, respectively). For *D. reticulatus*, activity showed pronounced peaks in autumn, with exceptionally high numbers of active ticks recorded in October–November. Females generally outnumbered males (1194 to 997 collected specimens) during major peaks, although both sexes followed broadly synchronous temporal patterns. In addition, smaller peaks were also observed in spring of subsequent years (Figure 2).

*I. ricinus* showed lower numbers of active specimens (females: 121; males: 146; nymphs: 155). Adult activity was most evident in late spring/early summer, while nymphs were abundant in the same seasonal window (Figure 2).

### 3.2. Effects of Seasonality and Weather Variables on Tick Activity

**Figure 3:**
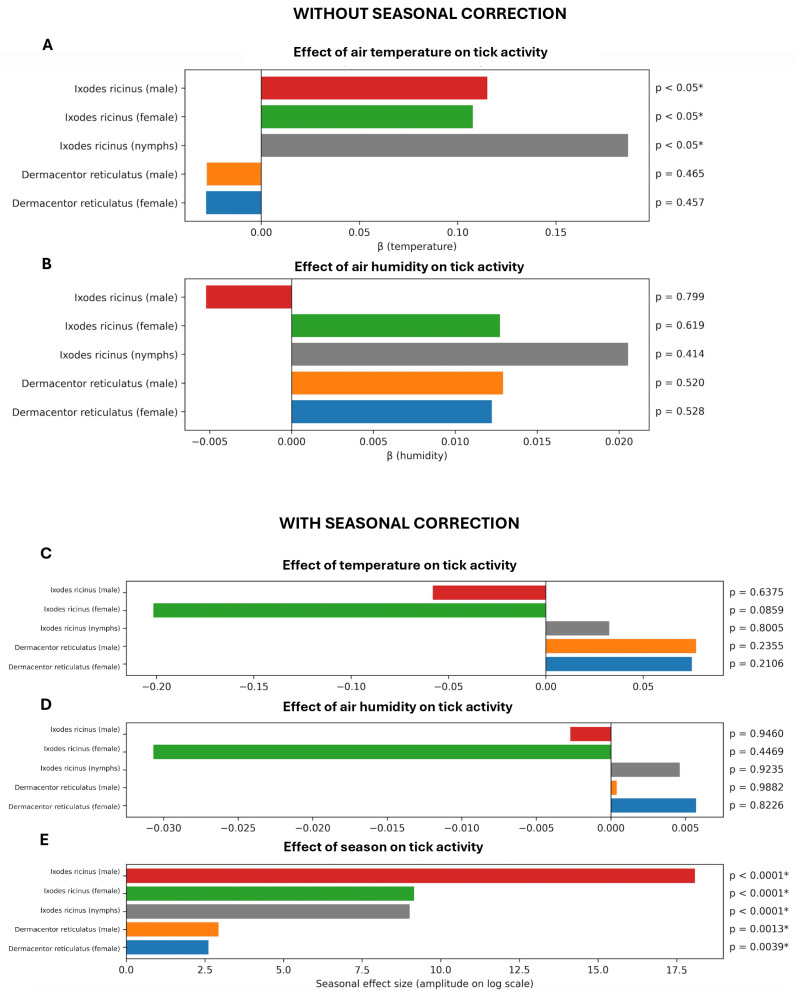
Effects of temperature, humidity and seasonality on tick activity. Panels (**A**,**B**) show the effects of temperature and humidity estimated using GAM negative binomial models without seasonal correction. Panels (**C**–**E**) present the corresponding models including seasonal effects. Results are shown separately for *Ixodes ricinus* (female, male, nymphs) and *Dermacentor reticulatus* (female, male). Bars represent regression coefficients (β) or seasonal effect size, and statistical significance was assessed using likelihood ratio tests. Asterisks indicate significant effects (*p* < 0.05).

The GAM-based modelling clarified that apparent weather effects in the uncorrected models largely reflect seasonal confounding. Without seasonal correction (Figure 3A,B), air temperature showed a positive association with the activity of *I. ricinus* males, females, and nymphs), with statistically significant effects (*p* < 0.05). In contrast, temperature effects for *D. reticulatus* adults were lower and not significant (*p* > 0.05). Importantly, relative air humidity did not show statistically significant effects on tick activity in the uncorrected models for either species/stage/sex (Figure 3).

After including seasonal correction (Figure 3C–E), the temperature effects became non-significant for all examined tick species and stages. Humidity remained non-significant after seasonal correction as well. Importantly, seasonality itself was strongly significant for all analysed tick categories (Figure 3E), with the largest seasonal effect sizes for *I. ricinus.*

### 3.3. Rodent-Associated Tick Infestation

Juvenile ticks were recorded on all examined rodent species; however, infestations were most frequently observed on *A. agrarius* with infestations involving both *I. ricinus* and *D. reticulatus* (Table 1, Supplementary Table S1). When larval and nymphal stages were analysed jointly, no statistically significant differences in infestation prevalence among rodent species were detected for either *I. ricinus* (χ^2^ = 4.31, df = 2, *p* = 0.115) or *D. reticulatus* (χ^2^ = 2.63, df = 2, *p* = 0.269). Although numerical differences in prevalence were observed among host species, these did not reach statistical significance.

Similarly, no significant difference in infestation prevalence was detected between male and female *A. agrarius* (*p* = 1.000).

## 4. Discussion

Our results show that across the five-year survey, *D. reticulatus* proved to be the overwhelmingly dominant tick species in the urban park, far outnumbering *I. ricinus* in questing collections (Figure 2). This finding is noteworthy because *I. ricinus* is generally considered the most widespread tick in Europe [2,27,28], yet our data echo reports from eastern Poland and adjacent regions where *D. reticulatus* can locally predominate [18,29].

The adults of *D. reticulatus* exhibited a striking seasonal activity pattern concentrated in autumn: we observed pronounced questing peaks in October–November, during which *D. reticulatus* densities surged to exceptionally high levels (Figure 2). These autumnal peaks consistently eclipsed the more modest spring upticks of *D. reticulatus* activity that occurred in some years. Interestingly, female of *D. reticulatus* tended to outnumber males at the height of the autumn peaks, although both sexes rose and fell in synchrony. In our opinion, this female-biased abundance during peak activity is likely related to the females’ urgent need to find hosts for engorgement (to ensure egg development). Overall, the strong autumn focus of *D. reticulatus* questing observed in our study aligns with previous multi-year observations, which often report a bimodal activity with a larger autumn peak and a secondary spring peak for this species [21,30,31].

By contrast, *I. ricinus* was present at much lower densities and showed a different seasonal pattern. *I. ricinus* adults were most active in late spring and early summer (primarily May–June), and we recorded nymphs predominantly in this same window (Figure 2). This corresponds to the well-known spring/early-summer peak of *I. ricinus* in temperate climates [32,33,34,35]. We did not observe a pronounced autumn peak for *I. ricinus* in our data—possibly because the hot, dry midsummer conditions in the open meadow habitat suppressed a later resurgence. Typically, in wooded habitats of Poland and Central Europe, *I. ricinus* exhibits a bimodal pattern with a major peak in late spring and a secondary peak in autumn [36]. The minimal autumn activity of *I. ricinus* in our study might reflect the habitat differences (open recreational grassland vs. forest microclimate) or simply stochastic variation in a low-density population. Nonetheless, the dominance of *D. reticulatus* over *I. ricinus* in an urban park is a remarkable outcome. It underscores how local habitat and microclimate conditions—in this case, a reclaimed post-industrial meadow with patchy shrubs—can favour one tick species to the near-exclusion of another. Open, grassy habitats are known to be preferred by *D. reticulatus*, whereas *I. ricinus* typically thrives in moist woodlands [22,37,38]. The study site’s characteristics (expansive meadow vegetation, invading goldenrod stands, and limited tree cover) likely created an ideal microhabitat for *D. reticulatus* (high sunlight for development, sufficient ground cover for questing and overwintering) while being less optimal for *I. ricinus* which requires higher sustained humidity [33,39]. This habitat-driven skew is consistent with regional observations that *D. reticulatus* dominates tick communities in eastern Poland [13,40]. It contrasts with Western European urban parks where *I. ricinus* is usually only or main tick species found [2,41,42].

To understand the drivers behind these seasonal patterns, we employed GAMs to test the influence of weather variables (temperature, humidity) versus intrinsic seasonal timing on tick activity. In models without a seasonal term, we found that air temperature had a positive association with *I. ricinus* activity—higher temperatures were linked to increased counts of *I. ricinus* females, males, and nymphs (with effects statistically significant at *p* < 0.05) (Figure 3). Interestingly, in these same initial models, temperature effects for *D. reticulatus* adults were much weaker and not significant (Figure 3), suggesting that *D. reticulatus* questing in our study was less sensitive to short-term temperature fluctuations—possibly because many *D. reticulatus* were active in the cooler autumn months anyway (Figure 2). In addition, relative air humidity alone did not show any significant effect on the activity of either species in the initial models (Figure 3). This lack of a standalone humidity signal might be due to the moderate range of humidity during our sampling (we avoided extremely dry days), or it may indicate that humidity thresholds for activity (e.g., saturation deficit limits) were rarely crossed in our sampling periods, at least not in a way detectable by linear models [43].

Crucially, when we incorporated a seasonal smoothing term (day-of-year) into the models to account for broad seasonal trends, the importance of weather variables changed (Figure 3). Seasonality itself, however, emerged as a strongly significant factor for all tick groups (with the largest seasonal effect sizes for *I. ricinus* nymphs and adults). These results suggest that the observed patterns of tick activity are driven predominantly by intrinsic seasonal dynamics (and possibly longer-term environmental cues correlated with season) rather than by immediate weather conditions on the day or week of sampling. In our opinion, tick population dynamics and questing phenology are largely regulated by seasonal developmental cycles and diapause that synchronize tick activity with favourable periods for host availability, rather than responding opportunistically to each weather change [44].

The host association data from rodent trapping provided complementary evidence about the ecology of tick maintenance in the park. We found that juvenile ticks (larvae and nymphs) of both species were present on all the small rodent species captured (Table 1, Supplementary Table S1). However, infestations were most frequently observed on *A. agrarius*, which appears to be a primary host for immature ticks in this habitat, and they carried both *I. ricinus* and *D. reticulatus* juveniles, sometimes concurrently on the same individual (Supplementary Table S1). This indicates a broad host affinity, i.e., *A. agrarius* is highly permissive to tick parasitism, making it an important reservoir host species for the local tick populations. Interestingly, when we pooled larvae and nymphs and compared infestation prevalence among rodent species, the differences were not statistically significant for either tick (no one rodent species had a significantly higher tick burden than another). This is likely due in part to sample size limitations or the opportunistic nature of tick–host contact; nonetheless, the numerical trend favoured *A. agrarius*. There was also no significant difference in tick infestation between male and female *A. agrarius*, suggesting that both sexes of this rodent are equally involved in supporting ticks. The prominence of *A. agrarius* as a tick host in our study is consistent with patterns observed in other European tick–rodent systems. *Apodemus* spp. (including *A. agrarius* and the wood mouse *A. sylvaticus* or *A. flavicollis*) are well-known as key hosts for larval and nymphal *I. ricinus* across Europe, particularly in peri-urban and rural interface areas [45,46,47,48].

Moreover, our study adds to growing evidence that immature *D. reticulatus* also utilize small rodents like *Apodemus* as their hosts (Table 1, Supplementary Table S1). We observed *D. reticulatus* larvae attached to *A. agrarius* underscoring that a robust rodent community in the park can support the full life cycle of the tick. The lack of host specificity at the rodent level—both *I. ricinus* and *D. reticulatus* immatures feeding on the same rodent individuals—also raises the possibility of co-feeding transmission of pathogens between the two tick species on shared hosts. While our study did not address pathogen infection in ticks, the host-sharing we observed suggests that the park’s ecology could facilitate such interactions. Importantly, *A. agrarius* is known to be a competent reservoir for some TBPs [49,50,51]. Thus, the strong association between juvenile ticks and *A. agrarius* in this urban site likely plays a role in maintaining pathogen cycles within the park.

Finally, our findings highlight some factors that might influence tick–host dynamics in this urban, post-industrial park. The dominance of *A. agrarius* among rodents may be a consequence of the habitat’s open and grassy character; *A. agrarius* is a species that thrives at forest-field edges and in overgrown fields, and it often colonizes peri-urban lots [52,53]. Its abundance would directly amplify tick reproduction by providing ample hosts for larvae and nymphs. In contrast, more forest-dependent rodents (like *A. flavicollis* or *Myodes* voles) were fewer, consistent with the sparse tree cover on site. The vegetation structure—notably the dense stands of Canadian goldenrod (*Solidago canadensis*) mentioned in the site description (Figure 1)—could also play a role. Goldenrod thickets may maintain higher humidity near the ground and provide shelter for rodents.

It is also worth considering potential hosts for adult tick stages in the studied urban environment, particularly in the context of their role as reservoirs and dispersal agents of TBPs. Due to the specific location of the park within a densely built-up urban area and its fenced character, the regular presence of large wild mammals as hosts for adult ticks appears largely excluded, although occasional incursions cannot be entirely ruled out. In this context, domestic animals, primarily dogs accompanying their owners during recreational activities may play a significant role as hosts for adult *I. ricinus* and *D. reticulatus* [54,55,56]. In addition, small- and medium-sized mammals inhabiting the area, such as hamsters and other synanthropic rodents, may sporadically serve as hosts for adult ticks [57]. The potential contribution of birds should also be considered, as some avian species frequent urban green spaces and may occasionally host adult ticks or facilitate their dispersal within and between urban habitats [58].

## 5. Conclusions

Our study demonstrates that urban recreational areas can sustain stable populations of medically and veterinary important ticks, constituting persistent foci of tick hazard. The tick community in the studied urban park was dominated by *Dermacentor reticulatus*, while *Ixodes ricinus* occurred at lower densities, indicating that local habitat structure influence species composition in urban environments.

Seasonal dynamics emerged as the primary driver of tick questing activity, whereas short-term variation in temperature and relative humidity played a subordinate role once seasonality was accounted for. Distinct seasonal activity patterns were observed between species and developmental groups, with *D. reticulatus* exhibiting pronounced autumn activity and *I. ricinus* peaking mainly in late spring and early summer.

Juvenile stages of both tick species were most frequently associated with *Apodemus agrarius*, suggesting that simplified rodent communities dominated by urban-adapted hosts are sufficient to sustain tick life cycles in urban green spaces. Collectively, these findings highlight the importance of seasonally structured tick activity and host availability in shaping tick hazard in urban environments.

From a practical perspective, the pronounced and predictable seasonal peaks in tick activity observed in this study suggest that preventive actions in urban recreational areas, such as public awareness efforts or targeted management measures could be timed to periods of highest risk rather than relying on short-term weather conditions.

## Figures and Tables

**Table 1:** Prevalence and mean intensity of tick infestation on rodents by tick species and stage. The table summarizes, for each rodent species, the number of examined and infested hosts, total number of collected ticks, prevalence of infestation, and mean intensity of infestation calculated separately for each tick species and developmental stage.

Host Species	Tick Species	Tick Stage	Hosts Examined (*n*)	Hosts Infested (*n*)	Total Ticks Collected	Prevalence (%)	Mean Intensity
*Apodemus* *agrarius*	*Dermacentor* *reticulatus*	L	51	9	73	17.6	8.1
		N	51	10	43	19.6	4.3
	*Ixodes ricinus*	L	51	15	48	29.4	3.2
		N	51	1	2	2.0	2.0
*Apodemus* *flavicollis*	*Dermacentor* *reticulatus*	L	4	2	7	50.0	3.5
		N	4	2	7	50.0	3.5
	*Ixodes ricinus*	L	4	2	7	50.0	3.5
		N	4	0	0	0.0	n/c
*Microtus* *arvalis*	*Dermacentor* *reticulatus*	L	2	0	0	0.0	n/c
		N	2	1	4	50.0	4.0
	*Ixodes ricinus*	L	2	0	0	0.0	n/c
		N	2	0	0	0.0	n/c

L—larvae, N—nymphs, *n*—number of counted specimens, n/c—no calculation performed.

## Data Availability

All data generated or analysed during this study are included in this published article and its Supplementary Materials.

## Supplementary Materials

The following supporting information can be downloaded at: https://www.mdpi.com/article/10.3390/biology15030252/s1, Table S1: Stage-specific and species composition of ticks collected from trapped rodents.

## Abbreviations

The following abbreviations are used in this manuscript:

TBDs	Tick-borne diseases
TBPs	Tick-borne pathogens
GAMs	Generalized additive models
LRTs	Likelihood ratio tests
DOY	Day of year

## References

[1] Diuk-Wasser M.A., Fernandez P., Vanwambeke S.O. (2025). Tick-Borne Diseases in Urban and Periurban Areas: A Blind Spot in Research and Public Health. Annu. Rev. Entomol..

[2] Hansford K.M., Wheeler B.W., Tschirren B., Medlock J.M. (2022). Questing *Ixodes ricinus* ticks and *Borrelia* spp. in urban green space across Europe: A review. Zoonoses Public Health.

[3] Janzén T., Choudhury F., Hammer M., Petersson M., Dinnétz P. (2024). Ticks—Public health risks in urban green spaces. BMC Public Health.

[4] Sormunen J.J., Kulha N., Klemola T., Mäkelä S., Vesilahti E.M., Vesterinen E.J. (2020). Enhanced threat of tick-borne infections within cities? Assessing public health risks due to ticks in urban green spaces in Helsinki, Finland. Zoonoses Public Health.

[5] Sormunen J.J., Kylänpää S., Sippola E., Elo R., Kiran N., Pakanen V.M., Kallio E.R., Vesterinen E.J., Klemola T. (2025). There Goes the Neighbourhood—A Multi-City Study Reveals Ticks and Tick-Borne Pathogens Commonly Occupy Urban Green Spaces. Zoonoses Public Health.

[6] Gray J., Kahl O., Zintl A. (2024). Pathogens transmitted by *Ixodes ricinus*. Ticks Tick-Borne Dis..

[7] Kubiak K., Szymańska H., Dziekońska-Rynko J., Tylkowska A., Dmitryjuk M., Dzika E. (2024). Tick-borne pathogens in questing adults *Dermacentor reticulatus* from the Eastern European population (north-eastern Poland). Sci. Rep..

[8] Szczotko M., Antunes S., Domingos A., Kubiak K., Dmitryjuk M. (2024). Tick-borne pathogens and defensin genes expression: A closer look at *Ixodes ricinus* and *Dermacentor reticulatus*. Dev. Comp. Immunol..

[9] Asman M., Bartosik K., Jakubas-Zawalska J., Świętek A., Witecka J. (2024). A New Endemic Locality of *Dermacentor reticulatus* in Central–Southern Poland and Its Potential Epidemiological Implications. Insects.

[10] Karbowiak G. (2022). Changes in the occurrence range of hosts cause the expansion of the ornate dog tick *Dermacentor reticulatus* (Fabricius, 1794) in Poland. Biologia.

[11] Dwużnik-Szarek D., Mierzejewska E.J., Kiewra D., Czułowska A., Robak A., Bajer A. (2022). Update on prevalence of *Babesia canis* and *Rickettsia* spp. in adult and juvenile *Dermacentor reticulatus* ticks in Poland (2016–2018). Sci. Rep..

[12] Kiewra D., Ojrzyńska H., Czułowska A., Dyczko D., Jawień P., Plewa-Tutaj K. (2025). *Dermacentor reticulatus* (Fabricius, 1794) in Southwestern Poland: Changes in Range and Local Scale Updates. Insects.

[13] Zając Z., Woźniak A., Kulisz J. (2020). Density of *Dermacentor reticulatus* ticks in eastern Poland. Int. J. Environ. Res. Public Health.

[14] Zając Z., Woźniak A., Kulisz J., Foucault-Simonin A., Obregón D., Moutailler S., Bartosik K., Cabezas-Cruz A. (2025). High prevalence of *Rickettsia* spp. among moderately dense populations of *Dermacentor reticulatus* ticks in south-central Poland. Med. Vet. Entomol..

[15] Zając Z., Kulisz J., Woźniak A., Obregón D., Foucault-Simonin A., Bartosik K., Moutailler S., Cabezas-Cruz A. (2024). Spatial distribution and pathogen profile of *Dermacentor reticulatus* ticks in southeastern Poland: A genetic and environmental analysis. Transbound. Emerg. Dis..

[16] Woźniak A., Zając Z., Kulisz J. (2025). Environmental Factors Driving the Seasonal Dynamics of *Ixodes ricinus* and *Dermacentor reticulatus* in Eastern Poland. Insects.

[17] Dyczko D., Kiewra D., Kolanek A., Błażej P. (2022). Influence of local environmental factors in southwestern Poland on the abundance of *Ixodes ricinus* and prevalence of infection with *Borrelia burgdorferi* s.l. and *B. miyamotoi*. Parasitol. Res..

[18] Zając Z., Obregón D., Foucault-Simonin A., Wu-Chuang A., Moutailler S., Galon C., Kulisz J., Woźniak A., Bartosik K., Cabezas-Cruz A. (2023). Disparate dynamics of pathogen prevalence in *Ixodes ricinus* and *Dermacentor reticulatus* ticks occurring sympatrically in diverse habitats. Sci. Rep..

[19] Elmieh N. (2022). The Impacts of Climate and Land Use Change on Tick-Related Risks.

[20] Yang X., Gao Z., Wang L., Xiao L., Dong N., Wu H., Li S. (2021). Projecting the potential distribution of ticks in China under climate and land use change. Int. J. Parasitol..

[21] Zając Z., Kulisz J., Woźniak A., Bartosik K., Khan A. (2021). Seasonal activity of *Dermacentor reticulatus* ticks in the era of progressive climate change in eastern Poland. Sci. Rep..

[22] Földvári G., Široký P., Szekeres S., Majoros G., Sprong H. (2016). *Dermacentor reticulatus*: A vector on the rise. Parasites Vectors.

[23] Taylor C.L., Lydecker H.W., Hochuli D.F., Banks P.B. (2023). Associations between wildlife observations, human–tick encounters and landscape features in a peri-urban tick hotspot. Urban Ecosyst..

[24] Rodriguez-Morales A.J., Shehata A.A., Parvin R., Tasnim S., Duarte P.M., Basiouni S. (2025). Rodent-Borne Parasites and Human Disease: A Growing Public Health Concern. Animals.

[25] Kaszewski B.M. (2008). Warunki Klimatyczne Lubelszczyzny.

[26] Estrada-Peña A., Mihalca A.D., Petney T.N. (2018). Ticks of Europe and North Africa: A Guide to Species Identification.

[27] Medlock J.M., Hansford K.M., Bormane A., Derdakova M., Estrada-Peña A., George J.C., Golovljova I., Jaenson T.G.T., Jensen J.-K., Jensen P.M. (2013). Driving forces for changes in geographical distribution of *Ixodes ricinus* ticks in Europe. Parasites Vectors.

[28] Perez G., Bournez L., Boulanger N., Fite J., Livoreil B., McCoy K.D., Quillery E., René-Martellet M., Bonnet S.I. (2023). Distribution, phenology, host range and pathogen prevalence of *Ixodes ricinus* in France. Peer Community J..

[29] Mierzejewska E.J., Welc-Faleciak R., Karbowiak G., Kowalec M., Behnke J.M., Bajer A. (2015). Dominance of *Dermacentor reticulatus* over *Ixodes ricinus* on livestock, companion animals and wild ruminants in Poland. Exp. Appl. Acarol..

[30] Bartosik K., Wiśniowski Ł., Buczek A. (2011). Abundance and seasonal activity of adult *Dermacentor reticulatus* in eastern Poland. Ann. Agric. Environ. Med..

[31] Olivieri E., Gazzonis A.L., Zanzani S.A., Veronesi F., Manfredi M.T. (2017). Seasonal dynamics of adult *Dermacentor reticulatus* in a peri-urban park in southern Europe. Ticks Tick-Borne Dis..

[32] Gray J.S. (2008). *Ixodes ricinus* seasonal activity: Implications of global warming. Int. J. Med. Microbiol..

[33] Schulz M., Mahling M., Pfister K. (2014). Abundance and seasonal activity of questing *Ixodes ricinus* ticks in their natural habitats in southern Germany in 2011. J. Vector Ecol..

[34] Egyed L., Élő P., Sréter-Lancz Z., Széll Z., Balogh Z., Sréter T. (2012). Seasonal activity and pathogen infection rates of *Ixodes ricinus* ticks in Hungary. Ticks Tick-Borne Dis..

[35] Zając Z., Kulisz J., Bartosik K., Woźniak A., Dzierżak M., Khan A. (2021). Environmental determinants of *Ixodes ricinus* occurrence and activity. Sci. Rep..

[36] Kubiak K., Dziekońska-Rynko J. (2006). Seasonal activity of *Ixodes ricinus* in forested areas of Olsztyn. Ann. Parasitol..

[37] Estrada-Peña A., Venzal J.M., Sánchez Acedo C. (2006). The tick *Ixodes ricinus*: Distribution and climate preferences in the western Palaearctic. Med. Vet. Entomol..

[38] Ehrmann S., Liira J., Gärtner S., Hansen K., Brunet J., Cousins S.A., Deconchat M., Decocq G., De Frenne P., De Smedt P. (2017). Environmental drivers of *Ixodes ricinus* abundance. BMC Ecol..

[39] Grigoryeva L.A. (2022). Influence of air humidity on survival and development of *Ixodes ricinus* (L., 1758) and *Ixodes persulcatus* Schulze, 1930 (Acari: Ixodidae). Syst. Appl. Acarol..

[40] Zając Z., Sędzikowska A., Maślanko W., Woźniak A., Kulisz J. (2021). Occurrence of *Dermacentor reticulatus* in the Wieprz River ecological corridor. Insects.

[41] Hansford K.M., McGinley L., Wilkinson S., Gillingham E.L., Cull B., Gandy S., Carter D.P., Vaux A.G.C., Richards S., Hayes A. (2021). *Ixodes ricinus* and *Borrelia burgdorferi* s.l. in the Royal Parks of London. Exp. Appl. Acarol..

[42] Cafiso A., Olivieri E., Floriano A.M., Chiappa G., Serra V., Sassera D., Bazzocchi C. (2021). Tick-borne pathogens in *Ixodes ricinus* in a peri-urban park in Italy. Pathogens.

[43] Kiewra D., Sobczyński M. (2006). Biometrical analysis of *Ixodes ricinus* in the Ślęża Massif. J. Vector Ecol..

[44] Perret J.L., Rais O., Gern L. (2004). Influence of climate on questing *Ixodes ricinus*. J. Med. Entomol..

[45] Tälleklint L., Jaenson T.G.T. (1997). Infestation of mammals by *Ixodes ricinus* in Sweden. Exp. Appl. Acarol..

[46] Boyard C., Vourc’h G., Barnouin J. (2008). Relationships between *Ixodes ricinus* and small mammals. Exp. Appl. Acarol..

[47] Michalik J., Hofman T., Buczek A., Skoracki M., Sikora B. (2003). *Borrelia burgdorferi* s.l. in *Ixodes ricinus* (Acari: Ixodidae) ticks collected from vegetation and small rodents in recreational areas of the city of Poznań. J. Med. Entomol..

[48] Karbowiak G., Miklisová D., Stanko M., Werszko J., Hajdul-Marwicz M., Szewczyk T., Rychlik L. (2019). The Competition Between Immatures of *Ixodes ricinus* and *Dermacentor reticulatus* (Ixodida: Ixodidae) Ticks for Rodent Hosts. J. Med. Entomol..

[49] Stefancíková A., Bhide M., Pet’ko B., Stanko M., Mosansky L., Fricova J., Derdáková M., Trávnicek M. (2004). Anti-*Borrelia* antibodies in rodents. Ann. Agric. Environ. Med..

[50] Hu C.M., Humair P.F., Wallich R., Gern L. (1997). *Apodemus* sp. rodents, reservoir hosts for *Borrelia afzelii* in an endemic area in Switzerland. Zentralbl. Bakteriol..

[51] Burri C., Schumann O., Schumann C., Gern L. (2014). Are *Apodemus* spp. mice and *Myodes glareolus* reservoirs for *Borrelia miyamotoi*, *Candidatus* Neoehrlichia mikurensis, *Rickettsia helvetica*, *R. monacensis* and *Anaplasma phagocytophilum*?. Ticks Tick-Borne Dis..

[52] Mitter G., Sumasgutner P., Gamauf A. (2015). Niche partitioning of *Apodemus* species in an urban environment. Ann. Des Naturhistorischen Mus. Wien. Ser. B Für Bot. Und Zool..

[53] Babińska-Werka J., Gliwicz J., Goszczyński J. (1979). Synurbization processes in *Apodemus agrarius*. Acta Theriol..

[54] Liberska J.A., Michalik J.F., Dabert M. (2023). Exposure of dogs and cats to *Borrelia miyamotoi*-infected *Ixodes ricinus* ticks in urban areas of the city of Poznań, west-central Poland. Ticks Tick-Borne Dis..

[55] Kocoń A., Nowak-Chmura M., Asman M., Kłyś M. (2023). Review of ticks attacking domestic dogs and cats, and their epidemiological role in the transmission of tick-borne pathogens in Poland. Ann. Agric. Environ. Med..

[56] Kocoń A., Asman M., Nowak-Chmura M., Witecka J., Rączka G. (2022). Exposure of domestic dogs and cats to ticks (Acari: Ixodida) and selected tick-borne diseases in urban and recreational areas in southern Poland. Sci. Rep..

[57] Hajdová B., Cellengova Z., Peťko B., Ondrejkova A., Lipinský J., Drážovská M. (2024). Tick population dynamics in the city of Košice (Eastern Slovakia): A public health study. Front. Ecol. Evol..

[58] Keve G., Sándor A.D., Hornok S. (2022). Hard ticks (Acari: Ixodidae) associated with birds in Europe: Review of literature data. Front. Vet. Sci..

