# Supplementary material for: Urban Recreation Areas as Foci of Tick Hazard: Multi-Year Seasonal Patterns of Ixodes ricinus and Dermacentor reticulatus Activity and Host Spectrum of Their Juvenile Stages in Eastern Poland
Source: Biology (Basel). 2026 Jan 29;15(3):252. doi: 10.3390/biology15030252 (PMC12896652; doi:10.3390/biology15030252)
Supplement: Supplementary file 1 [file biology-15-00252-s001.zip › biology-4086895-supplementary.pdf]

Supplementary Table S1. Stage-specific and species composition of ticks collected from trapped rodents

Number	Rodent			Collection date	*Dermacentor reticulatus*		*Ixodes ricinus*	
	Species	Sex	Weight		Larvae	Nymphs	Larvae	Nymphs
1	*Apodemus agrarius*	F	28,2	13.06	21	0	3	0
2	*A. agrarius*	F	19,8	13.06	17	0	0	0
3	*A. flavicollis*	M	21,1	26.06	4	0	0	0
4	*A. agrarius*	M	28,0	6.07	12	0	0	0
5	*A. agrarius*	M	26,0	6.07	0	0	0	0
6	*A. flavicollis*	F	28,9	6.07	0	4	5	0
7	*A. flavicollis*	F	19,5	26.07	0	3	0	0
8	*A. agrarius*	F	22,7	26.07	1	0	0	0
9	*A. agrarius*	M	21,0	10.08	3	1	0	0
10	*A. agrarius*	M	17,5	10.08	5	0	0	0
11	*A. agrarius*	F	16,1	10.08	7	0	0	0
12	*A. agrarius*	F	20,0	10.08	0	0	2	0
13	*Microtus arvalis*	F	21,0	10.08	0	4	0	0
14	*A. agrarius*	M	16,7	10.08	1	2	18	2
15	*A. agrarius*	M	13,5	21.08	6	1	3	0
16	*A. flavicollis*	F	15,7	21.08	3	0	2	0
17	*A. agrarius*	M	15,4	21.08	0	2	5	0
18	*A. agrarius*	M	15,2	21.08	0	0	0	0
19	*A. agrarius*	F	13,4	21.08	0	8	1	0
20	*A. agrarius*	F	15,9	21.08	0	0	0	0
21	*A. agrarius*	M	22,1	22.08	0	0	1	0
22	*A. agrarius*	F	15,2	22.08	0	8	4	0
23	*A. agrarius*	M	12,7	22.08	0	3	4	0
24	*A. agrarius*	F	15,3	22.08	0	2	1	0
25	*A. agrarius*	F	14,6	23.08	0	0	0	0
26	*A. agrarius*	F	12,3	23.08	0	5	0	0
27	*A. agrarius*	F	12,7	23.08	0	0	1	0
28	*A. agrarius*	M	12,8	23.08	0	0	0	0
29	*A. agrarius*	M	16,7	23.08	0	11	0	0
30	*A. agrarius*	F	14,3	23.08	0	0	0	0
31	*A. agrarius*	M	12,5	19.09	0	0	0	0
32	*A. agrarius*	M	12,7	19.09	0	0	0	0
33	*A. agrarius*	F	13,0	19.09	0	0	0	0
34	*A. agrarius*	M	27,5	19.09	0	0	0	0
35	*A. agrarius*	M	24,5	19.09	0	0	0	0

36	*M. arvalis*	M	10,7	19.09	0	0	0	0
37	*A. agrarius*	F	27,5	19.09	0	0	0	0
38	*A. agrarius*	M	24,7	19.09	0	0	1	0
39	*A. agrarius*	M	30,9	20.09	0	0	0	0
40	*A. agrarius*	M	19,1	20.09	0	0	2	0
41	*A. agrarius*	F	11,5	20.09	0	0	0	0
42	*A. agrarius*	M	24,7	20.09	0	0	0	0
43	*A. agrarius*	M	14,6	20.09	0	0	0	0
44	*A. agrarius*	M	16,6	20.09	0	0	0	0
45	*A. agrarius*	F	21,9	21.09	0	0	0	0
46	*A. agrarius*	F	13,4	21.09	0	0	1	0
47	*A. agrarius*	M	19,2	2.10	0	0	0	0
48	*A. agrarius*	M	15,2	2.10	0	0	0	0
49	*A. agrarius*	F	15,8	2.10	0	0	0	0
50	*A. agrarius*	M	15,0	2.10	0	0	0	0
51	*A. agrarius*	F	13,7	2.10	0	0	0	0
52	*A. agrarius*	F	12,9	2.10	0	0	1	0
53	*A. agrarius*	M	14,0	2.10	0	0	0	0
54	*A. agrarius*	M	15,8	3.10	0	0	0	0
55	*A. agrarius*	M	15,7	3.10	0	0	0	0
56	*A. agrarius*	F	14,9	3.10	0	0	0	0
57	*A. agrarius*	F	13,6	3.10	0	0	0	0

F – female; M - male
